# Effectiveness of a headache awareness campaign on behavioral change

**DOI:** 10.3389/fneur.2025.1572541

**Published:** 2025-04-25

**Authors:** Shinsuke Muraoka, Takumi Asai, Naoki Suzuki, Toshihisa Nishizawa, Kazuki Nishida, Basile Chrétien, Ryuta Saito

**Affiliations:** ^1^Department of Neurosurgery, Nagoya University Graduate School of Medicine, Nagoya, Japan; ^2^Department of Neurosurgery, Kariya Toyota General Hospital, Kariya, Japan; ^3^Division of Biostatistics, Department of Advanced Medicine, Nagoya University Hospital, Nagoya, Japan

**Keywords:** headache, migraine, medication overuse headache, behavioral change, headache awareness campaign, decision tree model

## Abstract

Headache is a significant public health issue due to its high prevalence, associated disability, and socioeconomic burden. In Japan, awareness of migraine prevention and the risks of medication overuse remains limited. This study examined whether increasing knowledge about headaches through an online educational campaign could lead to behavioral changes, such as more frequent visits to headache clinics and reduced misuse of headache medications. An online educational program on headaches was conducted for 1,829 hospital staff members, who first completed a questionnaire before watching an educational video. Six months later, they completed a second questionnaire to assess behavioral changes. The initial survey revealed that although headaches interfered with work and daily life for approximately 50% of participants, only 20% regularly sought medical care. The most common reason for not visiting a medical facility was reliance on over-the-counter medications. In the follow-up survey, 20% of participants had independently gathered information about headaches, 6% had visited a medical institution for a new headache, and 40% had reduced their use of painkillers after becoming aware of medication overuse headache (MOH). A decision tree model, using the reduction in painkiller use as the outcome, indicated that correct knowledge about migraine, including preventive treatments, and active information-seeking behavior were key factors in promoting behavioral change. These findings suggest that providing online headache education to hospital staff may contribute to improved headache management by increasing awareness of migraine prevention and MOH while reducing excessive painkiller use.

## 1 Introduction

Headache disorders pose a significant public health problem due to their high prevalence, associated disability, and socioeconomic impact. The World Health Organization (WHO) identifies tension-type headache, migraine, and medication-overuse headache as the most common neurological disorders (1). These conditions place a significant burden on both individuals and society. In Japan, eight million people suffer from migraines, resulting in an economic loss of three billion US dollars annually (2). In addition, migraines reduce productivity, with a study in North America showing that presenteeism leads to greater lost working hours than absenteeism (3).

Patients with chronic migraines reportedly experience four times more lost productivity than those with fewer headache episodes (4). Therefore, educating individuals on symptom management, self-management methods, and access to standard treatments is crucial. In recent years, calcitonin gene-related peptide (CGRP)-related drugs have been effective in migraine prevention (5–9); however, challenges in the treatment of migraine persist. Many headache sufferers rely on over-the-counter medications rather than seeking medical attention (10–12). Inappropriate use of over-the-counter medications and limited access to medical resources can lead to chronic migraines and medication-overuse headaches (MOH) (13–15). These can be prevented with appropriate prophylactic medications (7); however, patients rarely consult doctors. Clinicians are satisfied with simply ruling out urgent conditions related to headaches through imaging. There are also issues with inadequate diagnosis and treatment of primary headaches, as well as low patient satisfaction. Raising awareness about headaches and promoting appropriate medication use could potentially reduce the public health burden of headache disorders.

This study aimed to evaluate the effectiveness of an online headache awareness campaign targeting healthcare staff, specifically assessing changes in the daily use of pain medications and consultations with physicians for headaches.

## 2 Methods

A headache awareness campaign and survey were conducted at Kariya Toyota General Hospital from October to December 2023 using in-house terminals accessible to all 1,829 employees. The campaign and survey targeted all employees, including physicians, nurses, pharmacists, other medical staff, and administrative staff.

The campaign highlighted the differences between primary and secondary headaches, the general flow of headache care, diagnostic criteria for migraine, preventive treatment, acute migraine treatment, and medication-overuse headaches. Videos explaining these topics were created and distributed to all employees. After viewing the videos, employees were asked to complete an online survey (primary survey). Three months later, a follow-up survey was conducted to assess changes in attitudes toward headaches (secondary survey).

### 2.1 Statistical methods

All statistical analyses were performed using SPSS for Windows (version 20.0; IBM, Chicago, IL, United States). Responses to each question in the online survey were tabulated and compared using the chi-square test or Mann–Whitney U test, with statistical significance set at *p* < 0.05. Furthermore, a decision tree analysis was performed to assess predictive factors from multiple perspectives.

### 2.2 Ethical aspects

This study was conducted in accordance with the Declaration of Helsinki and was approved by the Institutional Review Board of Kariya Toyota General Hospital (approval number 1013, approval date 2024/6/14). After the analysis, to protect patient privacy, all identifiable personal data were removed from the database.

## 3 Results

**Figure 1:**
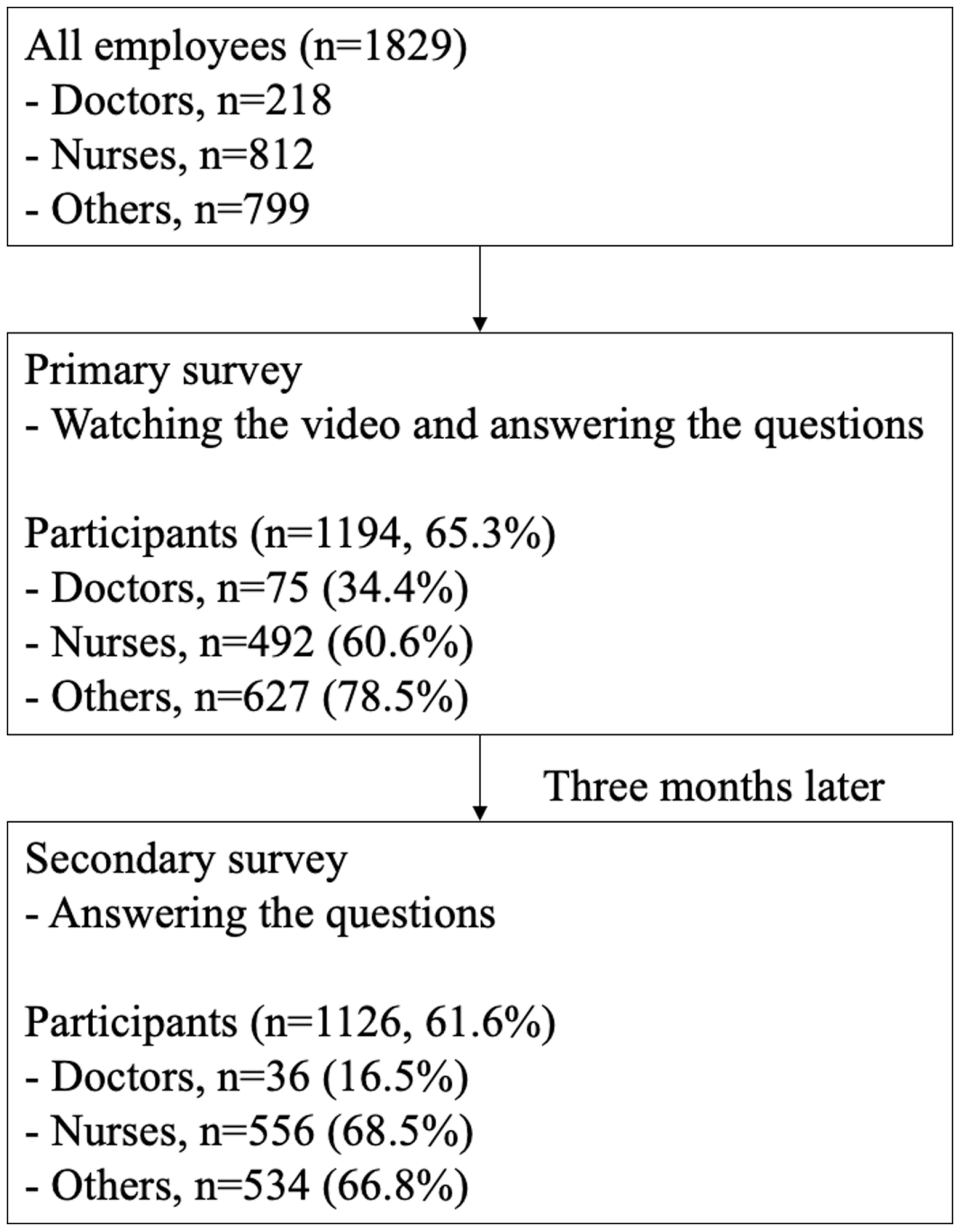
Study flowchart. At a regional core hospital with over 1,800 staff members, approximately 70% completed an online course on headaches.

As shown in Figure 1, of the total 1,829 participants in the primary survey, 1,194 participated in the primary survey, and 1,126 participated in the secondary survey. Among the respondents, 75 of 218 doctors (34.4%), 492 of 812 nurses (60.6%), and 627 of 799 other healthcare professionals (78.5%) participated.

### 3.1 Primary survey

**Figure 2:**
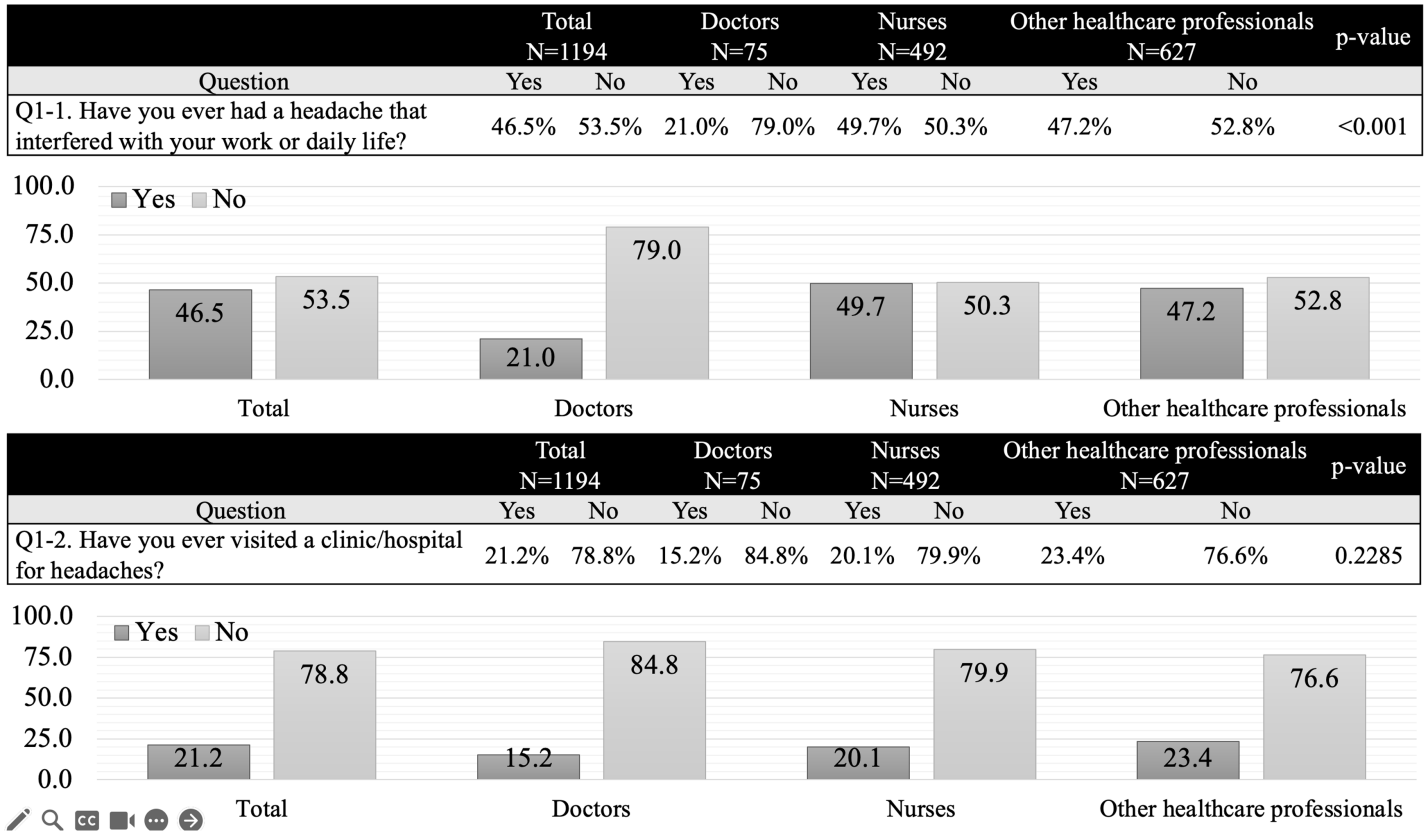
Primary survey. Compared to doctors, nurses and other healthcare professionals were more than twice as likely to experience headaches that interfered with work or daily life, with one in two reporting such issues. However, only approximately 20% had visited a medical institution for headaches.

**Figure 3:**
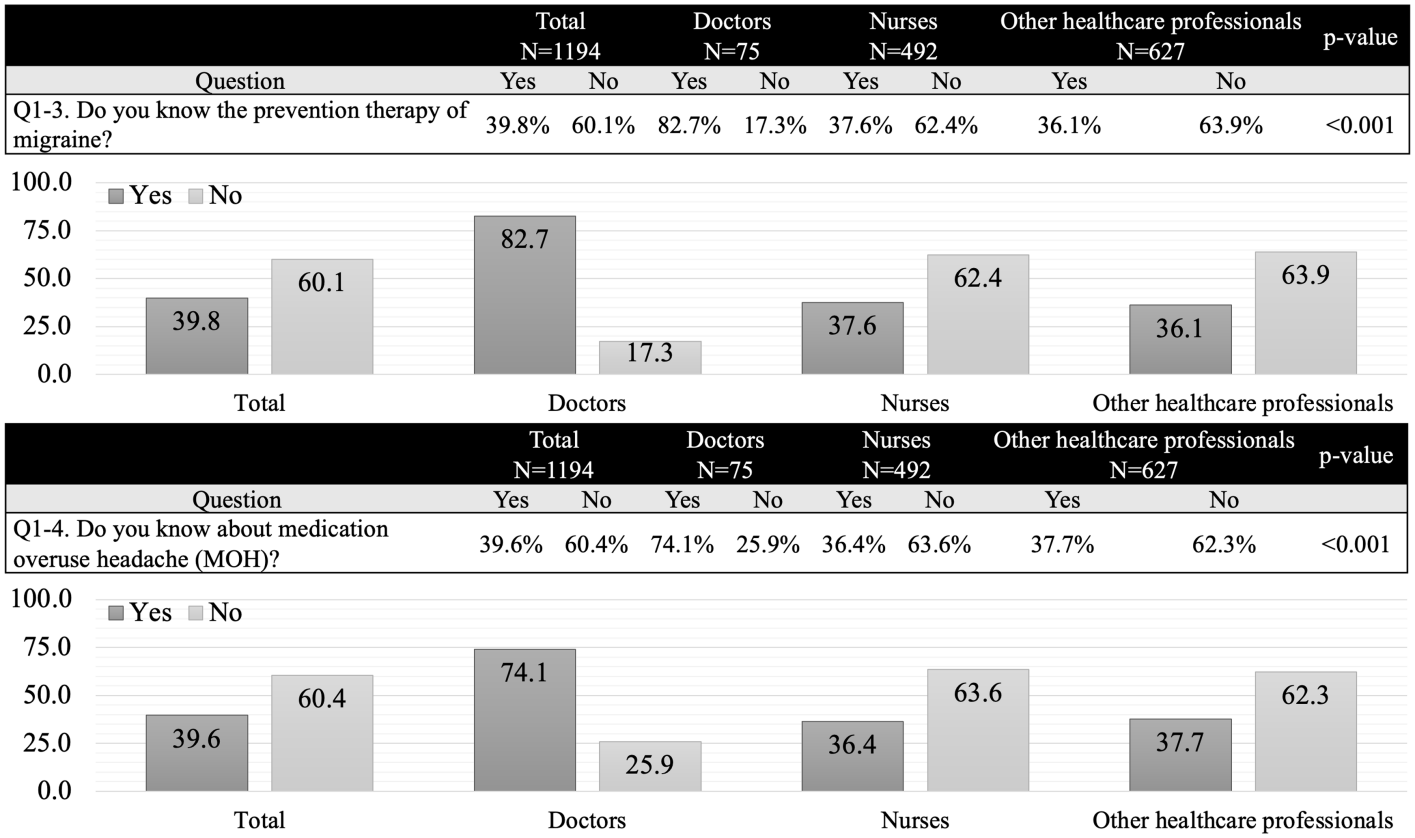
Primary survey. More than three-quarters of doctors were aware of preventive treatments for migraines and medication overuse headaches, whereas less than 40% of nurses and other healthcare professionals had similar awareness. These results suggest that nurses’ knowledge of headaches is comparable to that of other healthcare professionals.

After watching a video to gain general knowledge about headaches, participants completed the primary survey. Although 46.5% of respondents had experienced headaches that interfered with their work or daily life, only 21.2% had visited a clinic/hospital for their headaches (Figure 2). The most common reason for not seeking medical care was the belief that headaches could be managed with over-the-counter medications. A total of 388 participants (34.5%) were aware of how frequently they should consult a doctor for their headaches. Awareness of migraine prophylaxis and MOH was reported by 39.8 and 39.6% of respondents, respectively. Doctors demonstrated higher awareness of both migraine prophylaxis (82.7%) and MOH (74.1%) than nurses (37.6 and 36.4%, respectively) and other healthcare professionals (36.2 and 37.7%, respectively). Detailed findings are presented in Figure 3.

### 3.2 Secondary survey

**Figure 4:**
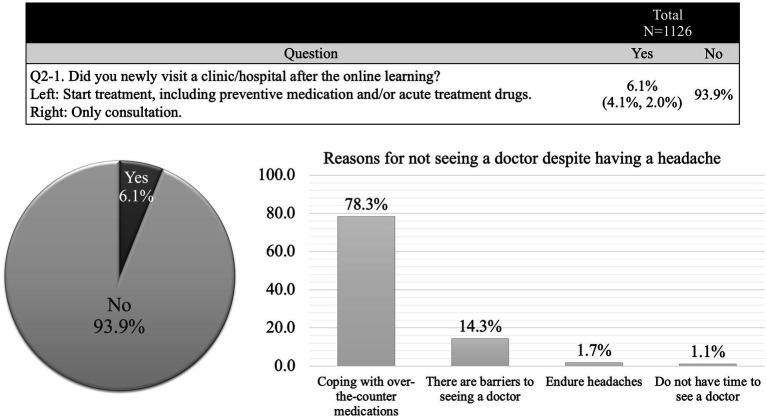
Secondary survey. Only 6% of participants visited a medical institution for the first time with headache as their primary complaint. The most common reason for not seeking medical care was reliance on over-the-counter medications to manage headache.

**Figure 5:**
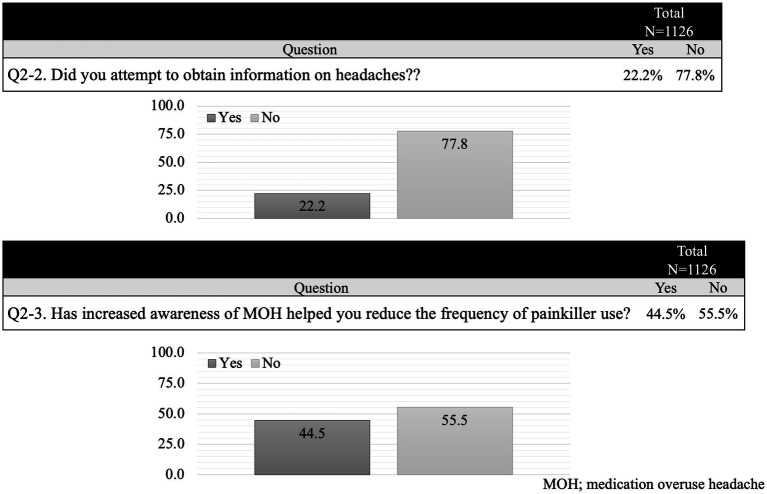
Secondary survey. After the first survey, 250 participants (22.2%) voluntarily collected information on headaches. Approximately half of the survey participants successfully reduced their frequency of painkillers.

A secondary survey was conducted 6 months after the primary survey. During this period, 250 participants (22.2%) voluntarily collected information on headaches, and 68 new participants (6.0%) visited a medical institution with headaches as their primary complaint. Among the 336 participants (70.6%) who did not visit a clinic/hospital—excluding the 553 who did not report any headache issues—the primary reason for not seeking care remained the same as in the initial survey. Additionally, 64 participants (13.4%) cited difficulties in visiting a clinic/hospital as a barrier (Figure 4). Following increased awareness of MOH, 501 participants (44.5%) reported a reduction in the frequency of painkiller use (Figure 5).

### 3.3 Decision tree model of predictive factors for the reduction of painkillers

A total of 805 participants were included in the decision tree analysis, which aimed to investigate the factors influencing the reduction in painkiller use following increased awareness of medication overuse headache (MOH). The primary decision node (Q2-3) assessed whether participants reported that increased awareness of MOH helped them reduce the frequency of painkiller use. Among them, 47% (*n* = 375) answered “Yes,” while 53% (*n* = 430) answered “No.”

For participants who did not perceive an impact of MOH awareness on their painkiller use (Q2-3 = No), further classification was performed based on their attempts to obtain information on headaches (Q2-2). Those who did not seek headache-related information (*n* = 158; 30%) were categorized as the least likely to reduce painkiller use. Conversely, those who attempted to obtain information were further stratified based on their knowledge of MOH (Q1-4). Participants who were aware of MOH (*n* = 102; 24%) were more likely to report a reduction in painkiller use compared to those who were not (*n* = 175; 42%).

Among participants who acknowledged the role of MOH awareness in reducing painkiller use (Q2-3 = Yes), subsequent classification was performed based on their knowledge of migraine prevention therapy (Q1-3). Those who were familiar with migraine prevention (*n* = 26; 4%) had the highest probability of reducing painkiller use, whereas those who were not (*n* = 90; 24%) underwent further classification based on MOH awareness (Q1-4).

**Figure 6:**
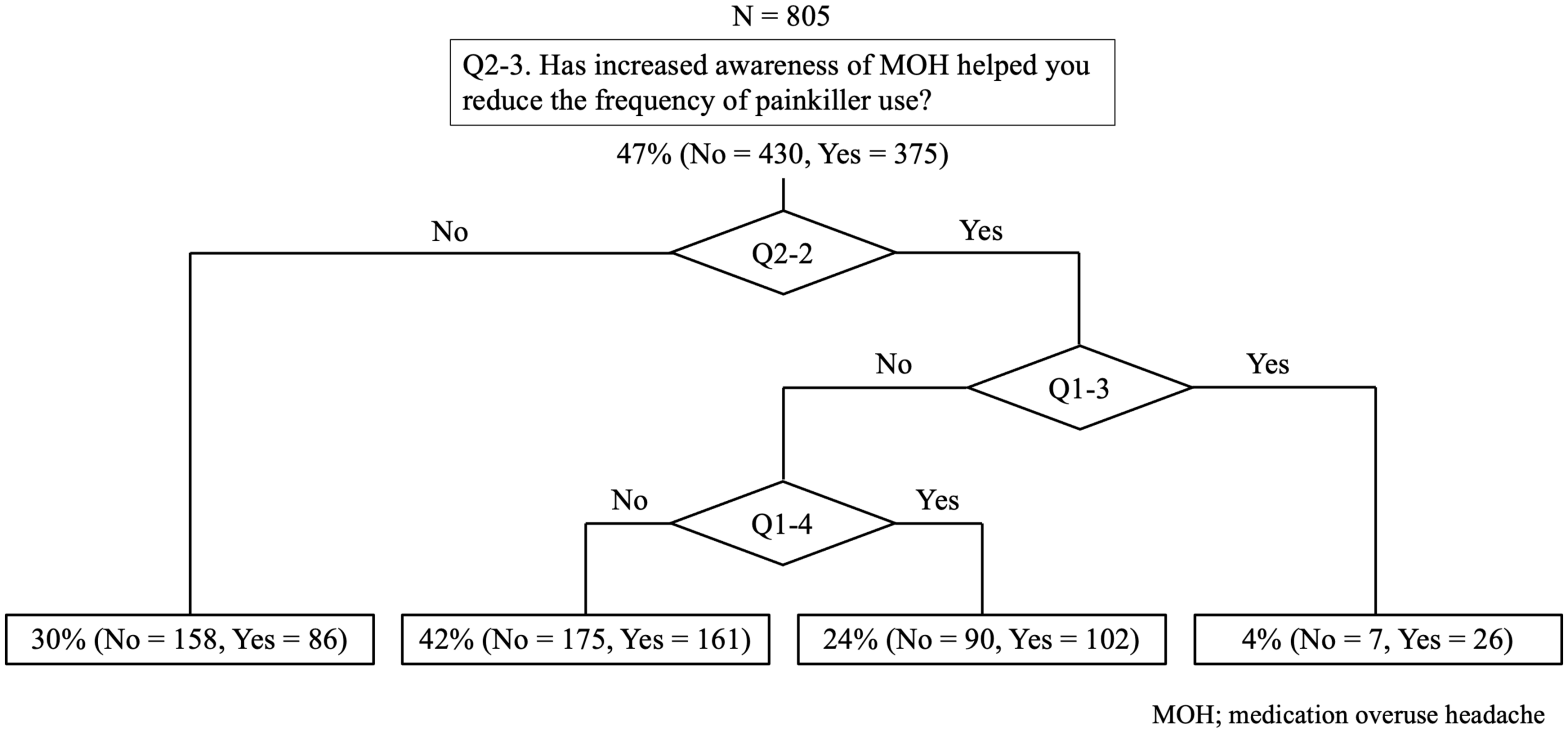
Decision tree model of predictive factors for the reduction of painkillers. Knowledge of migraine prevention therapy (Q1-3) had the strongest impact on reducing painkiller use. Awareness of medication overuse headache (MOH) (Q1-4) also influenced analgesic consumption, with informed individuals showing a greater likelihood of reduction. Additionally, actively seeking headache-related information (Q2-2) was associated with more appropriate painkiller use.

Overall, knowledge of migraine prevention therapy (Q1-3) and MOH (Q1-4) played crucial roles in determining painkiller use behavior. The group with the least likelihood of reducing painkiller use consisted of those who did not actively seek headache-related information (Q2-2 = No) or were unaware of MOH (Q1-4 = No) (see Figure 6).

## 4 Discussion

This headache awareness campaign suggests that providing online learning on headaches to all employees can enhance their understanding of headaches, encourage them to actively seek headache-related information, and increase their awareness of MOH. As a result, they may be more likely to refrain from overusing painkillers and increase the opportunity to newly visit to medical institutions.

### 4.1 Behavioral changes

The present study employed decision tree analysis to explore the impact of MOH awareness and related factors on the reduction of painkiller use among individuals experiencing headaches. The findings indicate that awareness of MOH alone is not sufficient to drive behavior change; rather, additional factors such as proactive information-seeking behavior and knowledge of migraine prevention therapy play a critical role in modifying analgesic consumption patterns.

The most significant predictor of painkiller use reduction was familiarity with migraine prevention therapy. Participants who were knowledgeable about preventive treatment strategies were the most likely to reduce painkiller consumption, highlighting the importance of patient education on migraine management beyond acute symptom relief. This finding aligns with previous studies suggesting that a comprehensive understanding of migraine pathophysiology and treatment options facilitates better self-management and adherence to appropriate therapies.

Additionally, MOH awareness contributed to a stratified effect, with those who were informed about MOH being more likely to alter their painkiller use than those who were not. This result underscores the necessity of raising awareness about MOH in clinical settings, as lack of knowledge may lead to continued medication overuse and worsening headache conditions.

Moreover, information-seeking behavior emerged as a key determinant of painkiller use modification. Participants who actively sought information on headaches demonstrated a higher probability of reducing their analgesic intake, suggesting that engagement with educational materials and healthcare providers may encourage more appropriate medication use. This finding highlights the potential value of digital health interventions, patient counseling, and targeted awareness campaigns in mitigating MOH-related medication overuse.

### 4.2 Headache awareness campaigns

The WHO’s cross-sectoral Global Action Plan was presented in 2024 (16), emphasizing the following:

1. Increasing policy priorities and strengthening governance for headache care
2. Providing effective and appropriate headache diagnosis, treatment, and care promptly
3. Implementing headache treatment and prevention strategies
4. Promoting headache research and innovation and strengthening information systems

To date, there have been various reports on headache awareness campaigns. In Denmark, an MOH public awareness campaign was implemented in 2016 (13), and has achieved high engagement with the general public, general practitioners, and pharmacists using various media. A 2014 MOH survey using social media among students receiving medical education at the University of Birmingham revealed that many respondents lacked knowledge about the MOH, and after being provided with information, less than 80% expressed an intention to reduce their use of painkillers (17). A recently successful awareness campaign was an in-house headache education program conducted by a Japanese IT company between 2019 and 2022 (18). This program involved 73,432 employees and revealed the prevalence of migraines, tension-type headaches, and cluster headaches. After the education, 82.9% of the participants said that their attitude towards colleagues with headaches had changed, and 72.5% said that their understanding of headaches had deepened. The awareness of the impact of headaches increased from 46.8 to 70.6%. Additionally, 4.1% expressed an interest in online consultations with headache specialists, many of whom had not previously received medical advice. This program led to an increase in productivity of approximately 14.7 days per year and a cost reduction of $4,531 per person, demonstrating the value of a migraine prevention program in the workplace.

In our survey, even among medical professionals, few had accurate knowledge of headache before the headache awareness campaign was implemented. In the second survey, conducted 3 months after the first, the number of people who visited a medical institution for headache increased slightly, and participants became more aware of the proper use of painkillers to prevent MOH.

### 4.3 The importance of early consultation

Migraine is a progressive disorder that increases in frequency and progresses to chronic migraine (19, 20). The main mechanisms of progression include changes in hypothalamic activity (21) and a decrease in the inhibitory effect of the brainstem (22). Factors that contribute to disease progression include frequency of attacks, excessive use of painkillers, comorbid pain syndromes, and obesity (19). Approximately 30% of patients with chronic migraine are resistant to prophylactic and acute treatments (23). CGRP-related drugs are effective for chronic migraine but are not as effective for episodic migraine (24–28). Therefore, early consultation and treatment are important before the migraines become chronic. Hirata et al. reported the consultation and treatment status of patients with migraines in Japan in 2021 (29). According to this study, 75.2% of patients used over-the-counter drugs for their headaches. In contrast, only 39.7% had visited a medical institution for migraines or severe headaches in the past year, and only 9.2% were taking preventive medications, which is very low. Buse et al. investigated the percentage of patients who received migraine prophylaxis in various countries. The percentages of patients who met the criteria for prophylaxis in the AHS GL2021 (30) were 54.1, 41.1, 51.9, and 59.1% in the United Kingdom, Germany, and Japan, respectively. However, the actual rate of patients receiving preventive treatment was 28.9% in the US, 21.2% in the UK, 20.8% in Germany, and 9.7% in Japan, showing that the rate of preventive treatment in Japan is lower than that in other countries (31).

In our survey, approximately half the respondents experienced headaches that interfered with their work. However, only approximately 20% had visited a medical institution with headache as the main complaint. It is important to continue providing correct information through head-awareness activities to prevent headaches from becoming chronic.

### 4.4 Medication overuse headache

MOH is a well-established cause of chronic daily headache, a term applied to patients with 15 or more headache days per month for > 3 months (32). It is estimated that the prevalence of MOH is 1–2% among the general population and can reach up to 50% among patients with chronic headaches (33). Katsuki et al. (34) conducted the first survey of the prevalence of MOH in Japan in 2022. They obtained 5,865 valid responses, and the prevalence of MOH was 2.32% (*n* = 136 cases) (35).

In our survey, less than 40% of the respondents were aware of MOH, and considering that the most common reason given for not visiting a medical institution with headache as the main complaint was that over-the-counter medication was sufficient, it was assumed that a certain number of people had or were developing MOH. However, in a survey conducted 3 months after the initial headache awareness campaign, just under half of the respondents said that they were now more aware of the number of times they used acute medication and could make an effort to reduce it in some cases. It is important to continue headache awareness campaigns, acquire correct knowledge, and perform preventative treatments as necessary.

### 4.5 Limitations

There are several limitations in this study. First, as the follow-up period was only 6 months and online video viewing was only performed for the first time, we believe that creating a system that provides more frequent e-learning over a long period is necessary. Second, the survey was conducted at in-house terminals. Because all employees had access to the terminals, there were no restrictions on accessing the questionnaire. However, as participation in the survey was voluntary, there was bias towards only those interested in headaches, particularly those who suffered from headaches. Therefore, we added questions about daily quality of life to encourage people without headaches to participate. However, even for medical professionals, the information does not reach those who are not interested in headaches. How to deal with this will be an issue for future research. Third, the exact number of individuals who had received preventive treatment before the first questionnaire is unknown. However, given that approximately 20% of participants visited medical institutions with headaches as their primary complaint and that 60% of non-physician staff were unaware of preventive treatments, the number of individuals receiving such treatment is expected to be low. Fourth, social desirability bias is presumed to exist. However, it has not been quantitatively assessed using the Marlowe-Crowne Social Desirability Scale or other methods. While anonymization is one approach to mitigating social desirability bias, in this study, it was essential to analyze the results of the first and second questionnaires together. Therefore, the data were collected without anonymization. Fifth, in this study, 805 participants responded to both the first and second surveys. While this can be interpreted as a positive trend for the group, the possibility that the analysis was insufficient in accounting for the temporal continuity of the data cannot be ruled out. Finally, this study did not include a control group and did not evaluate or compare the rate of behavioral change with or without online learning. Therefore, it does not precisely reflect the impact of a single online learning session. However, providing all employees with an opportunity to learn about headaches once, followed by a decrease in the frequency of painkiller use and an increase in first-time visits to a clinic/hospital, yielded highly valuable results.

## 5 Conclusion

Our campaign results emphasize the need for multifaceted patient education interventions that not only inform individuals about MOH but also provide guidance on migraine prevention strategies and encourage active patient participation in their own care. Healthcare professionals should consider integrating structured educational programs into routine headache management to enhance patient awareness, foster self-efficacy, and ultimately promote more effective treatment outcomes.

Further research should investigate longitudinal effects of MOH education and explore potential behavioral interventions that could reinforce sustained reductions in painkiller use. Additionally, future studies should assess whether personalized educational strategies tailored to patients’ baseline knowledge and information-seeking behaviors yield superior outcomes compared to generalized awareness campaigns.

## Data Availability

The raw data supporting the conclusions of this article will be made available by the authors, without undue reservation.
